# Developmental trajectory of guilt and shame during the transition to university

**DOI:** 10.3389/fpsyt.2025.1632419

**Published:** 2025-10-27

**Authors:** Chenglei Wang, Mingrui Zhang, Liangliang Chen, Zhaohua Tang, Chao Yan, Mengqing Long, Xinhua Yang

**Affiliations:** ^1^ Shanghai Changning Mental Health Center, Affiliated Hospital of East China Normal University, Shanghai, China; ^2^ School of Education, Hunan Agricultural University, Changsha, China; ^3^ School of Psychology and Cognitive Science, East China Normal University, Shanghai, China

**Keywords:** self-conscious emotions, guilt, shame, hopelessness, child maltreatment

## Abstract

**Background:**

The transition into adulthood is often accompanied by increases in negative self-conscious feelings and psychological distress. This study aimed to identify developmental trajectories in guilt and shame and their associations with psychological factors during the first university year.

**Methods:**

This cohort study examined changes in guilt and shame in a sample of first-year undergraduate students in China (n=311). Participants completed electronic surveys at the beginning, after two months and twelve months, with outcomes of guilt and shame, and predictors including childhood maltreatment, hopelessness, depression and suicidal ideation. A latent growth mixture model was used to analyze the developmental trajectories in guilt and shame, and the associations with potential risk factors were investigated with bivariate binary logistic regression models.

**Results:**

Two classes of guilt and shame trajectories were identified: the largest trajectory was decreasing in guilt whereas the most prevalent class was increasing for shame. Hopelessness was associated with the trajectory of both guilt and shame, whereas sexual abuse only predicted the increased trajectory of shame.

**Conclusions:**

These findings highlighted the different development trajectories and their distinct risk factors in guilt and shame, suggesting that the importance of distinguishing different constructs when studying negative self-conscious.

## 1 Introduction

The transition to university presents a unique opportunity for the self-conscious emotions development of guilt and shame (1). The main function of these emotions is to regulate social behavior by encouraging morally and socially acceptable behaviors, with a remarkable impact on interpersonal relationships and successful development (2). Individuals who experience guilt often feel regret and hope to alleviate their guilt through compensation behaviors, while individuals who experience shame may escape and hide when they perceive himself or herself deficient. However, when the experience of shame and guilt become pervasive, individuals are at risk for developing psychological problems including depression, suicide, and post-traumatic stress disorder (3). Given the transition to university coincides with the peak period for the onset of mental illnesses (4), it is thus critical to investigate the developmental trajectories of guilt and shame—distinct self-conscious emotions pivotal for social adaptation—to reveal dynamic patterns of risk and resilience obscured in cross-sectional data. It is essential that identifying how first-year college students experience these emotions.

Some people are more prone to experience guilt or shame than others. Tangney et al. developed a scenario-based self-report measure of guilt and shame that assesses these individual differences: the Test of Self-Conscious Affect (TOSCA) (2). The TOSCA recognizes the distinction between guilt and shame by centered on the role of the self. Based on this distinction, shame is an emotion linked with a focus on the global self, while guilt is an emotion arising in connection with specific behaviors in a specific context (2). In the measure, shame is fundamentally about the condemnation of a globally “bad” self, stimulating feelings of self-directed anger, efforts to hide from others and worthlessness. Guilt, by contrast, involves criticism of a specific act, not the self, usually combined with a plan or specific intention to remedy the problem, stimulating feelings of regret and a need for punishment. Research on the TOSCA has found that shame was related to poor psychological adjustment and depressive symptoms (5, 6). On the other hand, guilt was found to be either not correlated or negatively correlated with these symptoms (7) (8), suggesting that shame could be a more maladaptive emotion as compared with guilt. However, some researchers have argued that TOSCA might overestimate the maladaptive aspect of shame and underestimate that aspect of guilt (9). It should be noted that shame was not considered as a greater emotional source of psychological problems than guilt, instead the two emotions were a source of personal growth with adaptive or maladaptive outcomes (10).

Previous studies have found distinct changes pattern in these two emotions. In two longitudinal studies, shame decreased slightly over a 1 year period during early adolescence (11), whereas guilt increased over a period of six years from early childhood to early adolescence (12). Another cross-sectional study found shame decreased from adolescence into middle adulthood, reaching the minimum level around age 50, and then increased in old age. Whereas guilt increased from adolescence into old age, reaching a plateau at about age 70 (13), suggesting that people become more prone to experiencing adaptive self-conscious emotions (guilt), and less prone to experiencing maladaptive ones (shame). Indeed, emergent adulthood is a period of accelerated brain development, resulting in intensive self-conscious development and increased autonomy (1). However, literatures exploring the changes of guilt and shame over time are very small over the university transition period, little thus is known about whether there are sub-groups of individuals who experience similar patterns of change in these self-conscious emotions. Importantly, these findings have been mainly from individualistic Western cultures, and population heterogeneity in changes in the two emotions have not been fully investigated in collectivist Chinese cultures. Clarifying their developmental trajectories during this critical window is therefore essential to quickly identify individuals who deviate from the normative path and to inform targeted interventions.

Early adverse experiences may have lasting effects on people’s emotional responses including guilt and shame (14). Children exposed to an adverse childhood experience may develop a complex identification process whereby they accept responsibility for the caregiver’s neglect or abuse, thus resulting in guilt and shame to protect against a painful reality (15). A growing body of research provides consistent support for the positive relationship between childhood maltreatment and the two emotions, with various forms of maltreatment being linked with guilt and shame. For example, shame has positive associations between these in victims of childhood sexual abuse (16, 17). People who were sexually abused often describe themselves as damaged, unworthy, or insignificant, leading to heightened feelings of shame (18). This positive relationship was replicated between shame and emotional abuse or neglect. Individuals who experienced shame resulting from abuse or neglect perceived themselves as humiliation and worthlessness, could trigger feelings of helplessness and hopelessness, thus leading to an increased risk of depression and suicide (19). However, relations between child maltreatment and guilt have been examined less frequently, and when studied, inconclusive results were obtained. Specifically, guilt was positively associated with the experience of physical abuse but not emotional abuse/neglect (20). People with early traumatic experiences had a less increased sensitivity to guilt than shame in adulthood (21, 22). Theoretical models and prior research suggest that childhood trauma can damage the child’s self-schema, fostering core beliefs of worthlessness and a tendency for self-blame, which in turn predisposes individuals to maladaptive self-conscious emotions such as shame and excessive guilt, particularly under stress (23). It should be noted that these studies often focused on one or two specific types of maltreatment, without examining other forms of abuse and neglect, although different types of maltreatment often co-exist. It therefore remains unknown which types of abuse and neglect influence the development of guilt and shame in students during specific stages of life.

The present study aimed to investigate changes in guilt and shame among university students from entry to completion of first-year university. This investigation is informed by a conceptual framework positioning early maltreatment as a distal vulnerability factor, and proximal factors like hopelessness and depressive symptoms as manifestations of cognitive-affective distress that may precipitate maladaptive self-conscious emotions. Two research questions were examined: 1) How do the levels of guilt and self-blame change across the school year? Do the two emotions gradually decrease or increase? Do these self-conscious emotions have different developmental trajectories? 2) What explains the growth trajectories in guilt and shame for the sub-groups? Based on the literature and theoretical framework, we proposed the following specific and testable hypotheses:

H1: It was hypothesized that distinct latent classes would characterize the developmental trajectories of guilt and shame.
H2: The majority of students would follow a pattern of increase in shame whereas their guilt would show increased pattern.
H3: Childhood maltreatment, hopelessness and depressed mood would predict increase of both guilt and shame.
H4: Sexual abuse would be specific to the two emotions compared to other forms of child maltreatment.

## 2 Materials and methods

### 2.1 Participants

Participants were entering first-year undergraduate students from a public university in Hunan province who completed online questionnaires at three points during the 2018–2019 academic year. A total of 597 entering undergraduate students were eligible to participate in the study based on their required mental health education course. A total of 485 entering students chose to participate in the study (81%), provided complete data at baseline (September). The following exclusion criteria applied to all potential participants: 1) incomplete survey with missing data (n=9); 2) twenty-five participants repeatedly answered, so their second answers were excluded; and 3) completion time outliers: 144 participants were excluded because the completion time was shorter than 535 s (2.5th percentile) or longer than 3409 s (97.5th percentile). Applying these exclusion criteria, the initial sample (n=311) consisted of 257 females (79%) and 54 males (21%). The age range was 16 years old to 22 years old, with a mean age of 18.02 (*SD* = .80). Follow-up surveys were distributed by email at the end of the autumn term (December) and the beginning of the second year (September), which three time-points were as 0 months, 3 months and 12 months into the academic year. In total, 284 students completed the first follow-up survey, and 249 completed the second follow-up survey.

### 2.2 Measures

#### 2.2.1 Guilt and shame

The Chinese version of The Test of Self-Conscious Affect-III (TOSCA-III) consists of 15 brief scenarios likely to be encountered by adults in their daily lives (24). Each scenario is followed by a set of responses indicative of guilt, shame, externalization, detachment, and pride regarding the situation. Respondents are asked to rate the likelihood of engaging in each response using a 5-point scale (1 = not likely, 5 = very likely). To investigate guilt and shame, we did not make further use of externalization, detachment/unconcern and bride subscales in the present article. The Cronbach’s alpha coefficient of the two subscales at three different times were respectively.90,.94,.93.

#### 2.2.2 Hopelessness

The Chinese version of Beck Hopelessness Scale was a 20 item self-assessment instrument for the measurement of perceived helplessness (25). This scale consists of three factors: expectations of success, expectations of failure, and future uncertainty. The total score can range from 0 to 20, indicating the number of items endorsed in the hopelessness direction. At all three measurement waves, Cronbach’s alpha reliability estimates were sufficient (.79;.75;.79).

#### 2.2.3 Depressive symptoms

The Chinese version of Patient Health Questionnaire was a self-administered version for measuring the severity of depression (26). This scale contains 9 items, with the total score range from 0 to 27. The Cronbach’s alpha coefficient of the scale at three different times were respectively.89,.93,.93.

#### 2.2.4 Suicidal ideation

The Chinese version of Beck Scale for Suicide Ideation was used to measure the severity of suicidal wishes and plans (27). The scale consists of 19 items each answered on a 3-point Likert scale, with high total scores reflecting strong SI. Completion of the scale was stopped if participants scored 0 on items 4 and 5, and thus we had complete data for part 1. The Cronbach’s alpha coefficient of the scale at three different times were respectively.79,.68,.78.

#### 2.2.5 Child maltreatment

The Childhood Trauma Questionnaire-Short Form was used to assess child maltreatment experience at home (28). It is a 28-item retrospective self-report questionnaire with a 5-point Likert scale ranging from 1 (not at all) to 5 (very often), which is divided into five subscales: emotional and physical neglect, emotional and physical abuse, and sexual abuse. The Cronbach’s alpha coefficient of the scale at baseline was.72.

### 2.3 Procedure

Data were collected online using WeiJuanxing, a prevalent survey platform in mainland China. Informed consent was collected at the beginning of this study and participants were suggested to leave if they experienced any negative emotions during the survey. Participants were granted psychological course credit as compensation for participating. Research activities were reviewed by the Review Board of Hunan Agricultural University (2018118) and were part of a longitudinal study about anhedonia and suicidal risk.

### 2.4 Data analysis

Firstly, the patterns of missing data were examined, Full Info Max Likelihood (FIML) was used to lower the bias produced by missing data using Mplus 8.0. Longitudinal invariance of each assessment was conducted using a Multigroup Confirmatory Factor Analysis (MCFA), which helped to clarify whether the temporal change was attributed to true developmental change. A Latent Growth Mixture Modelling (LGMM) was used to investigate differences in longitudinal change among unobserved groups which were characterized by a different developmental process (growth trajectory) in guilt and shame. Multi-class models including random effects of slope and intercept were compared. Six measures of model fit were selected for the best trajectory shape: Akaike Information Criteria (AIC), Bayesian Information Criterion (BIC), sample size adjusted BIC(a-BIC), entropy, Vuong-Lo-Mendell-Rubin Likelihood Ratio Test (VLMR-LRT) and Bootstrap Likelihood Ratio Test (BLRT). In addition to statistical fit indices, each category needed to have no less than 5% of the total number of participants to ensure the reproducibility and stability of the results (31). Bivariate binary logistic regression analysis was used to modelling two binary dependent variables jointly as a function of covariates using R software. Two outcomes were trajectories of guilt and shame, potential predictors included demographics, five types of child maltreatment, hopelessness, depression and suicide ideation.

## 3 Results

### 3.1 Descriptive statistics

Across waves, shame scores (T1 = 45.32, T2 = 46.81, T3 = 47.51) increased over time in university students (F = 7.65, *p* = .001, η^2^ = .029), whereas guilt scores (T1 = 63.11, T2 = 60.73, T3 = 60.49) decreased (F = 14.49, *p* <.001, η^2^ = .054). Compared with males, females reported higher guilt (Mean Difference=3.908, *p* = .001, 95% CI = 1.61-6.20), however no group difference in shame was found.

### 3.2 Longitudinal measurement invariance

Measurement invariance found that the fit indexes of these two unconstrained models were good and met the preconditions for subsequent equivalence testing in **Table 1**. The CFI value declined less than 0.001, which indicated equivalent at different time points were consistent, thereby allowing testing the mean level development of guilt and shame by latent growth curve models.

**Table 1:** The model fit indices of measurement invariance.

Model	χ2	df	CFI	ΔCFI	TLI	ΔTLI	SRMR	RMSEA (90% CI)
Shame								
Configural invariance	1717.23	1029	0.805	_	0.786	_	0.073	0.051 (0.047, 0.056)
Metric invariance	1791.31	1059	0.792	-0.013	0.779	-0.007	0.083	0.052 (0.048, 0.056)
Scalar invariance	2023.57	1089	0.735	-0.057	0.726	-0.053	0.094	0.058 (0.054, 0.062)
Factor variance invariance	2025.78	1091	0.735	–	0.726	–	0.095	0.058 (0.054, 0.062)
Residual invariance	2164.03	1121	0.704	-0.031	0.703	-0.023	0.097	0.061 (0.057, 0.064)
Guilt								
Configural invariance	1672.23	1029	0.845	_	0.830	_	0.072	0.050 (0.045, 0.054)
Metric invariance	1692.12	1059	0.847	0.002	0.837	0.007	0.074	0.049 (0.044, 0.053)
Scalar invariance	1850.59	1089	0.816	-0.031	0.810	-0.027	0.080	0.053 (0.048, 0.057)
Factor variance invariance	1868.29	1091	0.812	-0.004	0.806	-0.004	0.096	0.053 (0.049, 0.057)
Residual invariance	2081.70	1121	0.768	-0.044	0.767	-0.039	0.111	0.058 (0.054, 0.062)

df, degrees of freedom; CFI, comparative fit index; TLI, Tucker-Lewis index; RMSEA, root mean square error of approximation; CI, confidence interval; SRMR, standardized root mean squared residual.

### 3.3 Guilt and shame trajectories

**Figure 1:**
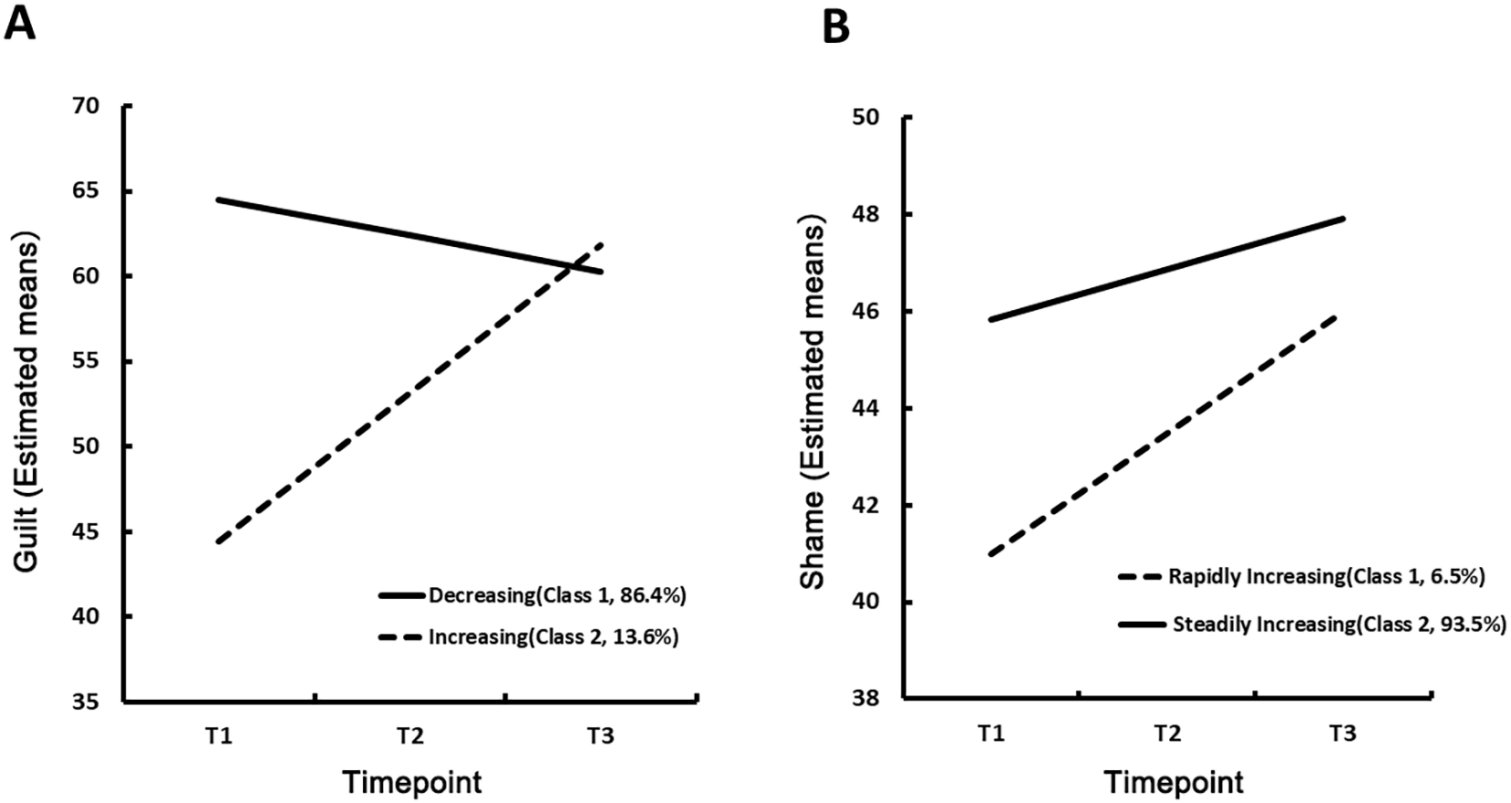
Two types of trajectories of guilt and shame. **(A)** The two-class solution for guilt. The largest group (86.4%) followed a decreasing trajectory, while a smaller group (13.6%) followed an increasing trajectory. **(B)** The two-class solution for shame. The majority of participants (93.5%) belonged to a stable increasing trajectory, and a minority (6.5%) to a rapid increasing trajectory.

Descriptive statistics for the variables and models fit indices are shown in **Table 2**. For shame, the three-class model showed relatively good fit to the data, as indicated by the lower AIC and BIC values and higher entropy values. However, the third category was less than 5% proportions of the total population. Thus, the two-class model was selected to be the optimal model for guilt: the largest group (86.4%) was in the decreasing trajectory and 13.6% were in the increasing trajectory (**Figure 1A**). Similarly, the two-class model was selected for shame: the largest group (93.5%) was in the stable increasing trajectory, 6.5% were in the rapid increasing (**Figure 1B**).

**Table 2:** Goodness of fit statistics for each class solution.

Variables	Model	AIC	BIC	a-BIC	Entropy	VLMR-MRT(*p*)	LMRT (*p*)	BLRT(*p*)	Smallest class (%)
Shame	1 class	3769.45	3797.46	3772.10	–	–	–	–	–
	2 classes	3758.65	3797.16	3762.29	0.83	0.012	0.016	<0.001	0.065
	3 classes	3759.03	3808.04	3763.66	0.88	0.471	0.484	0.364	0.013
Guilt	1 class	3672.66	3700.67	3675.31	–	–	–	–	–
	2 classes	3658.90	3697.41	3662.54	0.80	0.009	0.011	< 0.001	0.136
	3 classes	3654.34	3703.36	3658.98	0.87	0.026	0.031	0.094	0.008

AIC, Akaike information criterion; BIC, Bayesian information criterion; a-BIC, sample size-adjusted Bayesian information criterion; VLMR-LRT, Vuong-Lo-Mendell-Rubin likelihood ratio test; BLRT, bootstrapped likelihood ratio test.

### 3.4 Predictors of guilt and shame trajectories

Bivariate binary logistic regression analysis showed, compared to the increasing trajectory of guilt, the decreasing trajectory was negatively associated with hopelessness and being male. Compared to the rapid increasing trajectory of shame, the stable increasing trajectory was negatively associated with sexual abuse, and positively associated with hopelessness. Depression and suicidal ideation were not found to be significant association with the trajectories of guilt and shame, see **Table 3**.

**Table 3:** Bivariate binary logistic regression modeling of guilt and shame.

Variables	M ± SD	Guilt				Shame			
		Est	*OR*	95% CI	*p*	Est	OR	95% CI	*p*
Place of residence (country)	–	-0.22	0.80	-2.42, -1.97	0.70	-0.69	0.50	-3.17, -1.79	0.28
Gender (female)	–	-1.23	0.29	-3.09, -0.63	0.01	2.09	8.06	-2.44, -6.62	0.08
Suicide ideation	0.58 ± 1.31	-0.18	0.84	-1.04, -0.68	0.42	-0.14	0.87	-2.04, -1.76	0.78
Hopelessness	5.11 ± 3.61	-0.62	0.54	-1.58, -0.33	0.01	1.37	3.94	-1.04, -3.78	0.03
Depression	4.84 ± 4.03	0.36	1.43	-0.76, -1.49	0.22	0.75	2.11	-1.32, -2.82	0.16
Emotional abuse	7.01 ± 3.42	1.28	3.59	-2.77, -5.33	0.22	-0.39	0.68	-6.89, -6.11	0.82
Physical abuse	6.06 ± 3.02	-0.74	0.48	-4.23, -2.75	0.41	2.65	14.14	-5.88, -11.18	0.23
Sexual abuse	5.76 ± 2.81	-1.49	0.22	-5.52, -2.54	0.15	-3.78	0.02	-10.38, -2.82	0.03
Emotional neglect	12.68 ± 6.21	-0.06	0.94	-2.49, -2.36	0.92	-1.79	0.17	-5.62, -2.05	0.07
Physical neglect	9.31 ± 3.47	-0.30	0.74	-2.83, -2.23	0.65	1.44	4.23	-2.50, -5.39	0.16

Guilt (increasing = 1, decreasing = 2); Shame (stable increasing = 1, rapid increasing = 2).

## 4 Discussion

The present study investigated whether there are sub-groups who experience similar patterns of change in guilt and shame, and what predictors accounted for the sub-groups during the transition to university. Two different trajectories of guilt and shame were identified: most students experienced a decrease of guilt, whereas an increase of shame was felt by almost every student. The distinct predictors were found between the two emotions: being male and less hopelessness were associated with the decrease of guilt, whereas less sexual abuse and higher hopelessness were associated with the stable increase of shame. Our findings highlight how guilt and shame change as students enter into and engage with challenging learning environments over the school year.

The presence of sub-groups of individuals with different trajectories suggested population heterogeneity in two types of self-conscious emotion. A large number of university students demonstrated the decreasing trajectory of guilt and a steadily increasing trajectory of shame. Based on the TOSCA paradigm, guilt is associated with adaptive functioning whereas shame has been characterized as maladaptive and socially incompetent (9). Previous studies have found a sharp increase in psychological distress among first-year college students, with a plateau after this initial period of transition (29). Challenges faced by most first-year college students include managing increased academic demands, building new social networks, and navigating increased autonomy in activities of daily living (30). It therefore should not be surprising that these challengers increased maladaptive shame and decreased adaptive guilt among university students across the transition period. However, the finding is inconsistent with previous studies, with decreased shame and increased guilt during early adolescence (11) (12). A potential explanation is that shame might be sometimes experienced as a more socially-oriented emotion in this specific collectivist context compared to some individualistic cultures, potentially related to adapting to new social norms (32). In collectivist Eastern culture, shame is consistent with the values and norms endorsed by self-criticism and modesty that ultimately affirm social harmony (32). For building new social networks and getting involved with events, activities and sports on campus, it may be rather prominent to develop more external shame, which is related to how one perceives others’ thoughts of oneself (33), and can motivate students to take action in ways that are beneficial in their identity in a system of school connections. Thus, understanding the growth trajectories for the two subpopulations in guilt and shame is essential for choosing interventions during this period of rapid change and self-focus. Moreover, consistent with the prior study (34), individuals following the increasing guilt trajectory were more likely to be male. It indicated that under the specific stresses of the college transition (academic pressure, social challenges), a significant subset of male students may experience a pattern of escalating negative self-conscious emotions, particularly guilt.

Along with distinct trajectories of guilt and shame, another major finding was that guilt and shame had different predictors: high level of hopelessness predicted increase of both guilt and shame, whereas sexual abuse was specific to increase of shame but not guilt. According to attribution theory, the most common consequences of guilt and shame often coincide with negative feelings such as low self-esteem, embarrassment, and a fear of public humiliation (35). If one’s fault is traced back to uncontrollable and stable causes, guilt and shame will create a sense of helplessness and hopelessness. A recent study found self-blame were most strongly linked to hopelessness and worthlessness in Chinese university students and depressed patients (36). In clinical setting, depressed patients attribute blame to themselves in an overgeneralized way that is internal, global and stable, which results in them feeling helplessness, hopelessness, worthlessness, as well as guilty and depressed for their perceived failings (37). In an earlier retrospective study, a group of self-blaming feelings including self-disgust/contempt and guilt closely co-occur with feelings of inadequacy, hopelessness and depressed mood (38). These studies are consistent with our findings in first-year Chinese undergraduates, highlighted that hopelessness could play a key role in mental health problems, linked to guilt and shame. It suggests that cognitive intervention might be useful in preventing or reducing negative self-conscious and hopelessness in school psychological service.

Surprisingly, all types of child maltreatment, only sexual abuse experience had a positive association with rapid increase of shame in the current study. This is consistent with prior reports which stronger feelings of shame appeared to be positively related to an increase in those who had been sexually abused during childhood (39). In university samples, two studies have also found that sexual abuse entails more guilt and shame than other traumas do (40) and that the age when sexual abuse began may influence shame (41). According to attachment theory, adolescents react with sexual abuse to cope with overwhelming feelings of fear and sadness, especially when the perpetrator is a person with whom the adolescent has a close relationship (42). In the long-term, emotional numbing may have negative consequences such as withdrawal of empathy, maintenance of callousness toward others, and deficits in recognition of one’s own internal emotions, which then regulates shame, in turn determines the degree of stigmatization experienced (43). Such an explanation would be consistent with the results of the present study, indicating youth who experienced sexual abuse may experience mostly shame as a painful emotion with hostility initially directed towards the self, not guilt that motivates a repair of the perceived failure (44). The results of present study are additional clues that highlight the association between childhood sexual abuse and feelings of shame in young adulthood.

Contrary to expectations, a positive relationship between child maltreatment and guilt in the present study was not revealed. Previous studies proposed that children who were raised in households where they were made to feel that they had done something wrong, had something to hide, or were responsible for problems might be left with lingering feelings of guilt (19). However, the results of earlier studies also suggested stronger relationships of childhood trauma with shame than guilt (45), or indicated that it was associated only with shame but not with guilt (46), or even found that guilt was more linked to warm and supportive parental parenting (47). Failure to demonstrate this relationship in the present study may be also due to the specifics of the measure of shame that was used, which the TOSCA underestimates the maladaptive aspect of guilt and overestimate that aspect of shame (9). Earlier studies using the TOSCA also did not reveal relations between adverse childhood experiences and guilt (21). Thus, it could be that the TOSCA does not capture guilt-related feelings experienced by victims of childhood abuse.

This study provided strong empirical support guilt and shame as distinct emotions with different antecedents, experiences, and consequences (48). The fact that they have opposing trajectories over a stressful life transition, which call for university counseling center to assess them separately rather than viewing as a monolithic problem. The increasing trajectory of shame is a major red flag, indicating a deepening negative core self-evaluation. Interventions must focus on diffuse from shame-based thoughts and help break the cycle of avoidance and internalization (49). Students reporting high shame could be advised to join workshops on hope-building or cognitive-behavioral skills groups before severe shame consolidates. Since hopelessness predicted negative trajectories for both the two emotions, it should be a primary target in psychological screening during the first semester. The unique link between sexual abuse and increased shame highlights that shame, more than guilt, may be the primary emotional pathway which lead to long-term psychopathology through specific traumas. University services should provide specialized trauma therapies (50) that directly address shame and self-blame are essential to prevent the chronic and severe psychological outcomes.

The present study had several limitations. Firstly, it should be noted that the TOSCA measures maladaptive aspects of shame and adaptive aspects of guilt. Other measures such as the Guilt and Shame Proneness Scale focus on guilt and shame feelings but not compensatory actions could be useful in providing a full picture of how guilt and shame differ and function among Chinese people (51). Secondly, although our sample was reasonably representative of the student population, female was over-represented, results may still not be generalizable to all college students. Additionally, the second survey was sent out prior to final exams, which is a stressful time for students. As this is a stressful time for students, it may have resulted in a lower response rate and have impacted the levels of internalizing symptoms reported. Finally, all measures relied on self-report questionnaires rather than clinical interviews, which were dependent on individuals’ subjective reports and could have potential reporting bias. Further replication in other samples, especially in clinical samples, is needed.

## 5 Conclusions

In conclusion, the present study showed different developmental trajectories of guilt and shame, providing a more holistic picture of students’ self-conscious of guilt and shame across their time at university. Gender, hopelessness and childhood maltreatment seem to be differentially related to guilt versus shame in young adult. This research has potential implications for university mental health policies designed to improve the wellbeing of first-year students by providing a better understanding of how self-conscious emotions are experienced by certain groups of students.

## Data Availability

The raw data supporting the conclusions of this article will be made available by the authors, without undue reservation.
